# Biomarkers of myocardial injury in rats after cantharidin poisoning: Application for postmortem diagnosis and estimation of postmortem interval

**DOI:** 10.1038/s41598-020-69118-4

**Published:** 2020-07-21

**Authors:** Youyou Zhang, Yalei Yu, Jie Zhang, Chuhuai Guan, Liang Liu, Liang Ren

**Affiliations:** Department of Forensic Medicine, Tongji Medical College, Huazhong University of Science and Technology, Wuhan, 430030 China

**Keywords:** Biomarkers, Cardiology

## Abstract

Postmortem diagnosis of cantharidin-induced myocardial injury and postmortem interval estimation (PMI) are the challenges in forensic science. Cardiac biomarkers play an important role in the prediction and diagnosis of myocardial injury and can be used to determine the PMI. Based on the evidence, we aimed to explore the biomarkers which may be used for the postmortem diagnosis of cantharidin-induced myocardial injury and PMI estimation using the study of the proteins expression of TN-T, VEGF and HIF-1α by ELISA. Results of this study suggested that postmortem pathological changes were difficult to identify due to the autolysis of myocardium 72 h after death in cantharidin poisoning group. The plasma levels of TN-T and HIF-1α/TN-T are cardiac biomarkers with higher diagnostic accuracy for postmortem diagnosis of cantharidin-induced myocardial injury, VEGF/HIF-1α promises to be a biomarker for PMI estimation. Further studies are needed to verify these biomarkers, based on population, for being a useful tool in postmortem diagnosis of cantharidin-induced myocardial injury and PMI estimation.

## Introduction

Cantharidin poisoning is uncommon but fatal in clinical practice^1^, especially in patients who irrationally used it for treatment^2,3^ or as an aphrodisiac^3–5^ and patients of accidental poisoning due to blister beetle ingestion^6–8^. In our previous study, we found the myocardial injury induced by cantharidin is one of the most serious organ damages and depends on the dose and duration of exposure^3^. However, many patients have no obvious poisoning symptoms at the early stage of cantharidin poisoning and most of them have poor prognoses once diagnosed as acute circulatory failure after cantharidin poisoning. In the postmortem diagnosis of myocardial injury after poisoning, conventional postmortem diagnostic monitoring makes use of pathological examination and toxicological analysis, which requires either biopsy or necropsy specimens. what is more, the histopathological changes were not found in some cases of acute poisoning or the results of toxicological analysis showed that the the poison content is far less than fatal concentration. Concerned with this issue, in the very early stage of cantharidin poisoning, negaitive pathological evidence and toxicological analysis can be detected in cases of sudden death. Consequently, a profitable method to resolve difficulties was the detection of cardiac biomarkers which may predict organ damages. And the application of biomarkers in the postmortem diagnosis of acute myocardial damage caused by cantharidin poisoning will be provide an objective basis for decision making in forensic idientification work.

Estimation of postmortem interval (PMI) is one of the challenges in forensic science, especially in the poisonous cases, the homicidal cases of cantharidin poisoning are much inconspicuous, which have brought quite difficulty to crack the criminal case and the analysis of the PMI is the key to hunting for clue. And previous studies showed that cardiac biomarkers can be used to determine the PMI^9–11^. Overall, it is essential to explore the biomarkers which may be used for the postmortem diagnosis of cantharidin-induced myocardial injury and PMI estimation in cantharidin poisoning.

Troponin T (TN-T) is the major biomarker in the clinical diagnosis of acute myocardial injury^12^, the use of TN-T in the postmortem diagnosis of acute myocardial infarction and sudden cardiac death have been proved by some previous studies^13,14^. Previous findings suggest that cardiac troponins are specific markers of myocardial damage and elevated postmortem blood TN-T levels may be indicative of the severity of myocardial damage from various causes of death at the time of death^15^. What is more, many previous studies have evaluated the potential forensic use of TN-T to determine the PMI in deaths of different causes^16^. However, the sensitivity and specialty of TN-T in the postmortem diagnosis of myocardial injury and the relationship between TN-T and PMI estimation are controversial^13–18^. So that it is necessary to verify whether the TN-T can be used for the postmortem diagnosis of cantharidin-induced myocardial injury and explore possible serum biomarkers which can be used for postmortem diagnosis and PMI estimation. The results of our previous study on the pathological changes of cantharidin-treated rat hearts showed that ischemia and hypoxia were the main changes^19^. Through the literature retrieval, we found that the expression of vascular endothelial growth factor (VEGF) and hypoxia inducible factor-1α (HIF-1α) were related to the ischemic and hypoxic changes in myocardium. Previous studies showed that hypoxia is a stimulator for VEGF expression and HIF-1α is a major regulator for oxygen homeostasis^20,21^; What is important, HIF-1α pathway and VEGF pathway are the important mechanisms during the hypoxia or ischemia have been proved by previous studies^22,23^. So that the expression of HIF-1α and its downstream gene VEGF may play an important role in the changes of hypoxia and ischemia related to cantharidin poisoning. And we found that the expression of VEGF and HIF-1α were positively related with TN-T and myocardial demage area in our previous study^24^. Based on these evidences, we aim to evaluate the role of TN-T, VEGF and HIF-1α in postmortem diagnosis of cantharidin-induced myocardial injury and PMI estimation in this study.

To explore the biomarkers of myocardial injury in rats after cantharidin poisoning, we observed the postmortem pathological changes of rat hearts and detected the expression of TN-T, VEGF and HIF-1α in rat serum at different postmortem intervals, followed by analyzing the postmortem diagnostic adequacy of TN-T, VEGF and HIF-1α and the relationship between biomarkers and postmortem interval in our present study.

## Methods

### Experimental animals and protocols

Cantharidin was purchased from Shanghai Aladdin Bio-chem Technology Co., Ltd (Shanghai China) and suspended in sodium carboxymethyl cellulose solution by ultrasound. Sprague–Dawley (SD) rats weighing 200–230 g were maintained on a 12-h light/dark cycle in a controlled temperature (20–22 °C) and humidity (40%-50%) environment, which had unlimited access to food and water. Rats were acclimatized to the animal facility for a week and then used for next study. All animal protocols were performed in accordance with the guide for the care and use of laboratory animals published by the US National Institutes of Health (NIH Publication No. 85–23, revised 1996) and were approved by the Huazhong university of science and technology animal welfare committee.

### Animal models used for postmortem diagnosis and PMI studies

Fifty-four male SD rats were randomly divided into control group (n = 24) and experimental group (n = 30). The rats in control were sacrificed by execution after administration under 3% pentobarbital, and rats in experimental group were administrated cantharidin (4 mg/kg) suspension by method of intragastric and all rats died spontaneously within 6 h. Then, all death rats were stored in an environment with constant temperature and humidity (20 °C temperature, relative humidity of 50%). The rats in each group were randomly divided into six groups based on the time of heart sample and cardiac blood collection: 6 h, 12 h, 24 h, 48 h, 72 h and 168 h after death (n = 4 in control group and n = 5 in experimental group).

### Hematoxylin–eosin(HE ) staining

HE staining was conducted according to the routine protocols. The steps are as follows: after deparaffinization and rehydration, 4 μm longitudinal sections were stained with hematoxylin solution for 5 min followed by few seconds in 1% acid ethanoland and rinsed in distilled water. Then the sections were stained with eosin solution for 2 min and followed by dehydration with graded alcohol and clearing in xylene. The mounted slides were then examined and photographed using microscope after drying^24^.

### Enzyme linked immunosorbent assay (ELISA)

The rat TN-T ELISA kit (JYM0646Ra), rat VEGF ELISA Kit (JYM0634Ra) and rat HIF-1α ELISA Kit ( JYM0271Ra) were purchased by Wuhan colorful gene biological technology Co., ltd (Wuhan China). The procedure for the determination of TN-T, VEGF, HIF-1α concentration in rat serum was followed by the Sandwich-ELISA method^24^.

### Data analysis

Results are shown as mean ± SEM. Multiple comparisons between groups were analyzed by ANOVA. Correlation analysis used Pearson correlation analysis, ROC curve was used to evaluate the postmortem diagnostic adequacy, and linear regression analysis used regression statistics. A value of *P* < 0.05 was considered significant. Statistical analysis was performed using SPSS19.0.

## Results

### Pathological changes of rat hearts

**Figure 1:**
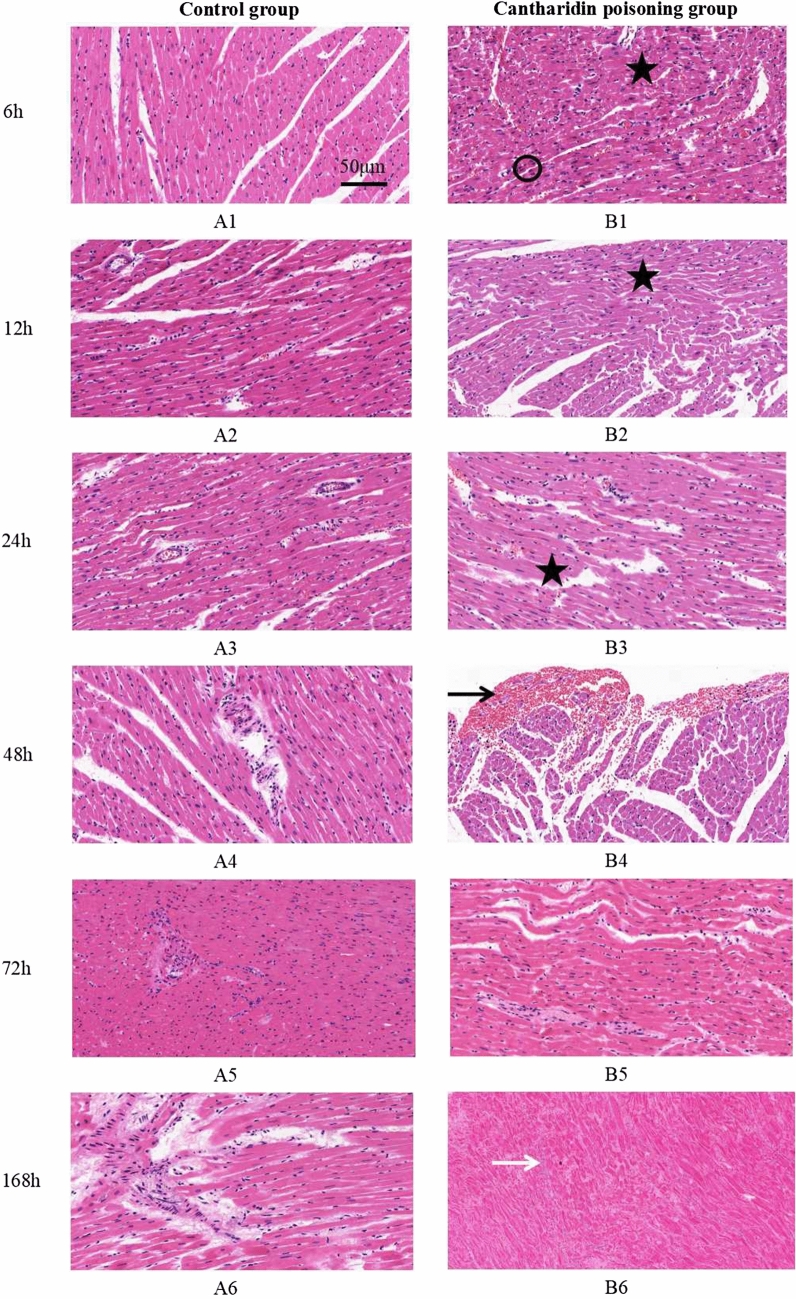
Postmortem pathological changes of rat hearts (bar = 50 μm, at 200 × magnification). (**A1**–**A6)** The postmortem pathological changes of rat hearts in control group and no abnormality was found in control group besides the change of cytoplasm dissolution in myocardium; (**B1**–**B6**) The postmortem pathological changes of rat hearts in cantharidin poisoning group, eosinophilic increased in cytoplasm (black cycle), degeneration, necrosis and the fusion of focal degeneration in myocardium (black asterisk), hemorrhagic foci in cardiac muscle (black arrow) and infiltration of inflammatory cells in myocardium (white arrow).

Figure 1 showed the postmortem pathological changes of rat hearts in different time intervals of control group (Panel A1-A6) and cantharidin poisoning group (Panel B1-B6). Cytoplasm dissolution was found in all groups, unclear transverse striation of myocardial fiber was found in group of 48 h and 72 h in control, the nuclear were karyolysis and disappeared in 168 h in control. Myocardial contraction band necrosis, inflammatory cell infiltration and hemorrhage in the subendocardial were observed in group of 6 h, 12 h, 24 h, and 48 h in cantharidin poisoning group while these changes were difficult to identify in group of 72 h and 168 h. Panel B-6 shows the nucleus is completely dissolved and the cell structure disappears in the hearts of cantharidin-treated rats.

### Postmortem changes of TN-T, VEGF and HIF-1α

**Figure 2:**
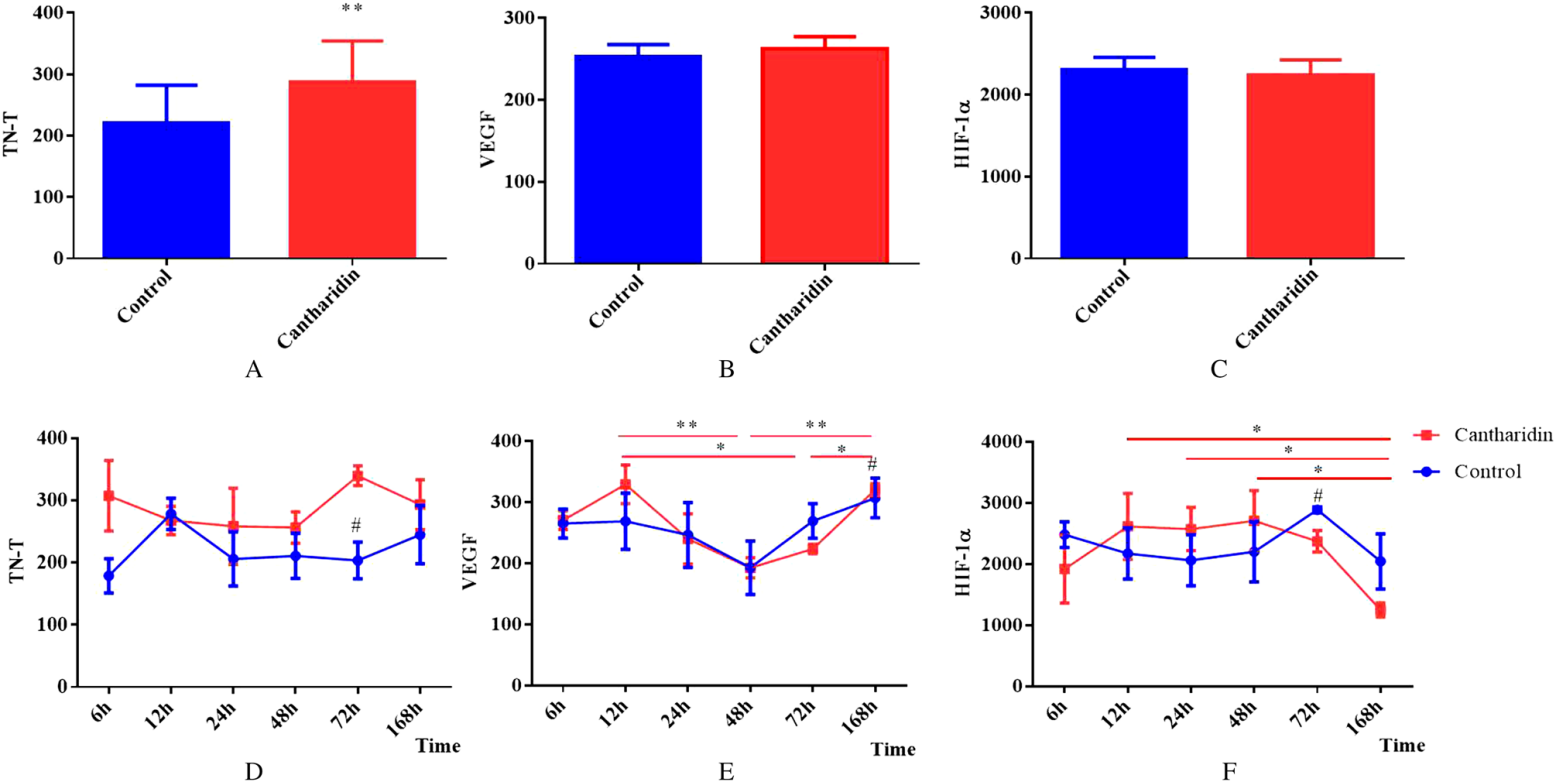
Postmortem changes of TN-T, VEGF and HIF-1α (**, *P* < 0.01; *, *P* < 0.05; #, compared with the cantharidin poisoning group, *P* < 0.05). (**A**–**C**) The difference of the postmortem expression of TN-T, VEGF and HIF-1α between the control group and cantharidin poisoning group respectivly; (**D**–**F**) The postmortem changes of TN-T, VEGF and HIF-1α in each time interval both in control and in cantharidin poisoning group respectively.

Firstly, we analyzed the total expression of TN-T, VEGF and HIF-1α in all time intervals between control group and experimental group, Fig. 2A showed that the TN-T expression in experimental group was significantly increased compared with the control (*P* = 0.004), and we did not observe any significant differences between the two groups in expression of VEGF (Fig. 2B) and HIF-1α (Fig. 2C). Then we further analyzed the trends of postmortem changes of TN-T, VEGF and HIF-1α; The expression of TN-T showed a increase tendency in experiment compared with the control in each time interval and statistically significant difference was found in group of 72 h (*P* = 0.015); And the TN-T expression in each time interval between the groups pointed to no tendency of changes with time. (Fig. 2D). Figure 2E showed that the postmortem expression of VEGF in control was stable, while statistically significant differences were found in expermental group and the VEGF expression in group of 48 h and 72 h were all significantly decreased compared with the group of 12 h, 168 h respectively (all *P* value < 0.05); We also found that the VEGF expression in experimental was significantly increased in the time interval of 168 h after death compared with the control (*P* = 0.035). And VEGF expression both in control and experimental presented on trends in consistency, which reduced first then increased, and the valleys of them were at the time interval of 48 h (Fig. 2E). The postmortem HIF-1α expression in control was also stable, but statistically significant differences were found respectively between the group of 168 h and 12 h, 24 h, 48 h in the experimental (all *P* value < 0.05); Moreover, the HIF-1α expression in experimental was significantly increased in the group of 72 h after death compared with the control (*P* = 0.046); The postmortem changes of HIF-1α both in control and experimental trended to stability within 72 h, then after 72 h experienced the process of descending. (Fig. 2F).

### Postmortem changes of VEGF/TN-T, HIF-1α/TN-T and VEGF/HIF-1α

**Figure 3:**
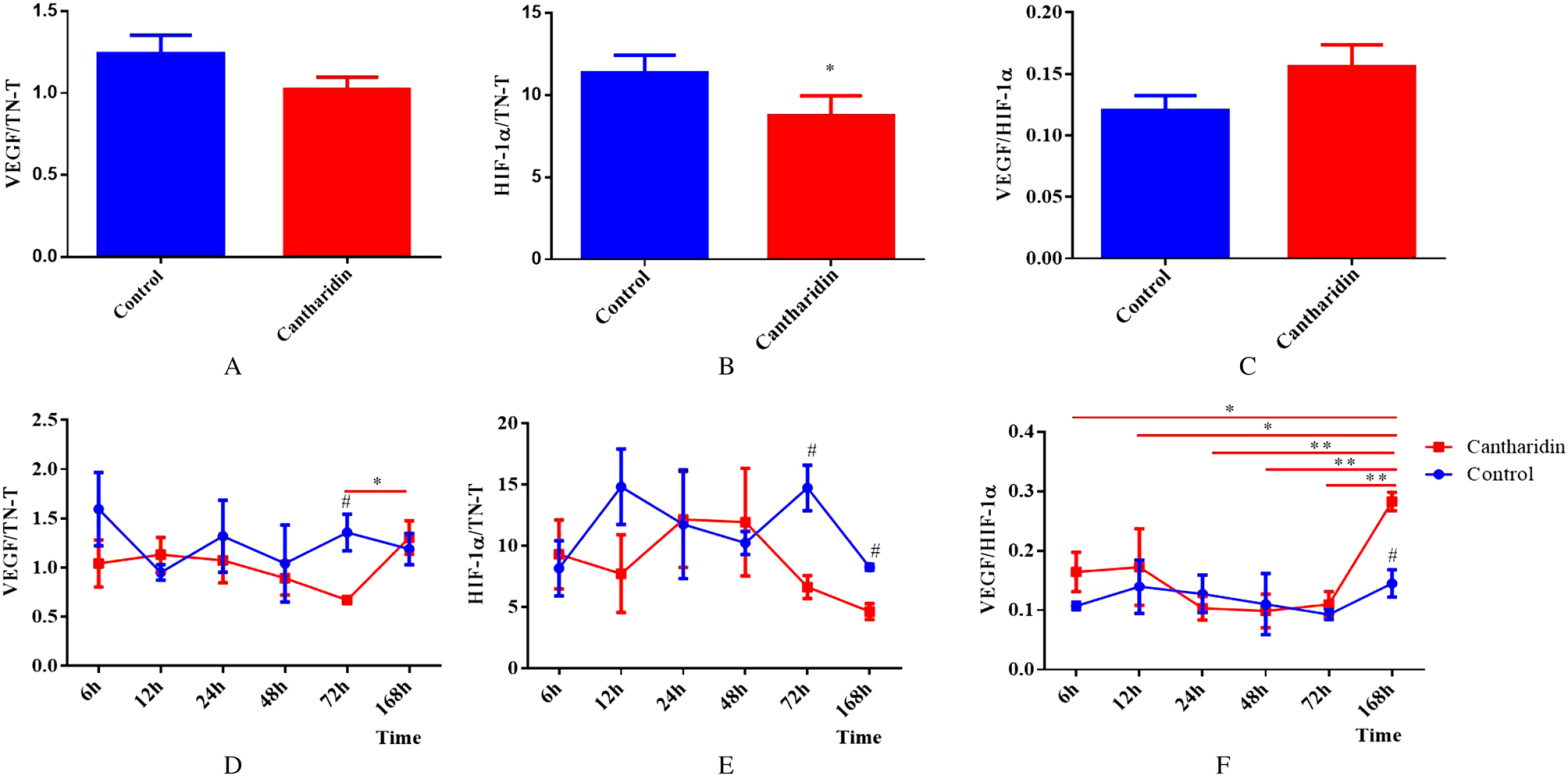
Postmortem changes of VEGF/TN-T, HIF-1α/TN-T and VEGF/HIF-1α (**, *P* < 0.01; *, *P* < 0.05; #, compared with the cantharidin poisoning group, *P* < 0.05). (**A**–**C**) The difference of the ratios of VEGF/TN-T, HIF-1α/TN-T and VEGF/HIF-1α between the control group and cantharidin poisoning group respectivly; (**D**–**F**) The postmortem changes of the ratios of VEGF/TN-T, HIF-1α/TN-T and VEGF/HIF-1α in each time interval both in control and in cantharidin poisoning group respectively.

We tried to get more information for postmortem diagnosis of cantharidin-induced myocardial injury using the data obtained from our study, the ratios of VEGF/TN-T, HIF-1α/TN-T and VEGF/HIF-1α were analyzed and showed in Fig. 3. Figure 3A indicated that the ratio of HIF-1α/TN-T was significantly decreased compared with the control (*P* = 0.024) while the ratios of VEGF/TN-T (Fig. 3B) and VEGF/HIF-1α (Fig. 3C) with no statistical difference between the two groups. Figure 3D–F showed that the tendency of postmortem changes of VEGF/TN-T, HIF-1α/TN-T and VEGF/HIF-1α in control were stable; And in experimental group, the ratio of VEGF/TN-T was stable within 72 h but the statistically significant difference (*P* = 0.007) was found between the group of 72 h and 168 h (Fig. 3D); The ratio of HIF-1α/TN-T was also stable among the all time intervals in experimental (Fig. 3E); Fig. 3F indicated that the ratio of VEGF/HIF-1α was stable within 72 h, and the ratio of VEGF/HIF-1α in group of 168 h was significantly increased compared with the other time intervals (all *P* value < 0.05). We further analyzed the changes of the ratios in each time interval between the groups and the statistical differences (all *P* value < 0.05) were found in the group of 72 h on the ratio of VEGF/TN-T and HIF-1α/TN-T; What is more, the statistical differences (all *P* value < 0.05) were also found in the group of 168 h on the ratio of HIF-1α/TN-T and VEGF/HIF-1α between the two groups. (Fig. 3D–F).

**Figure 4:**
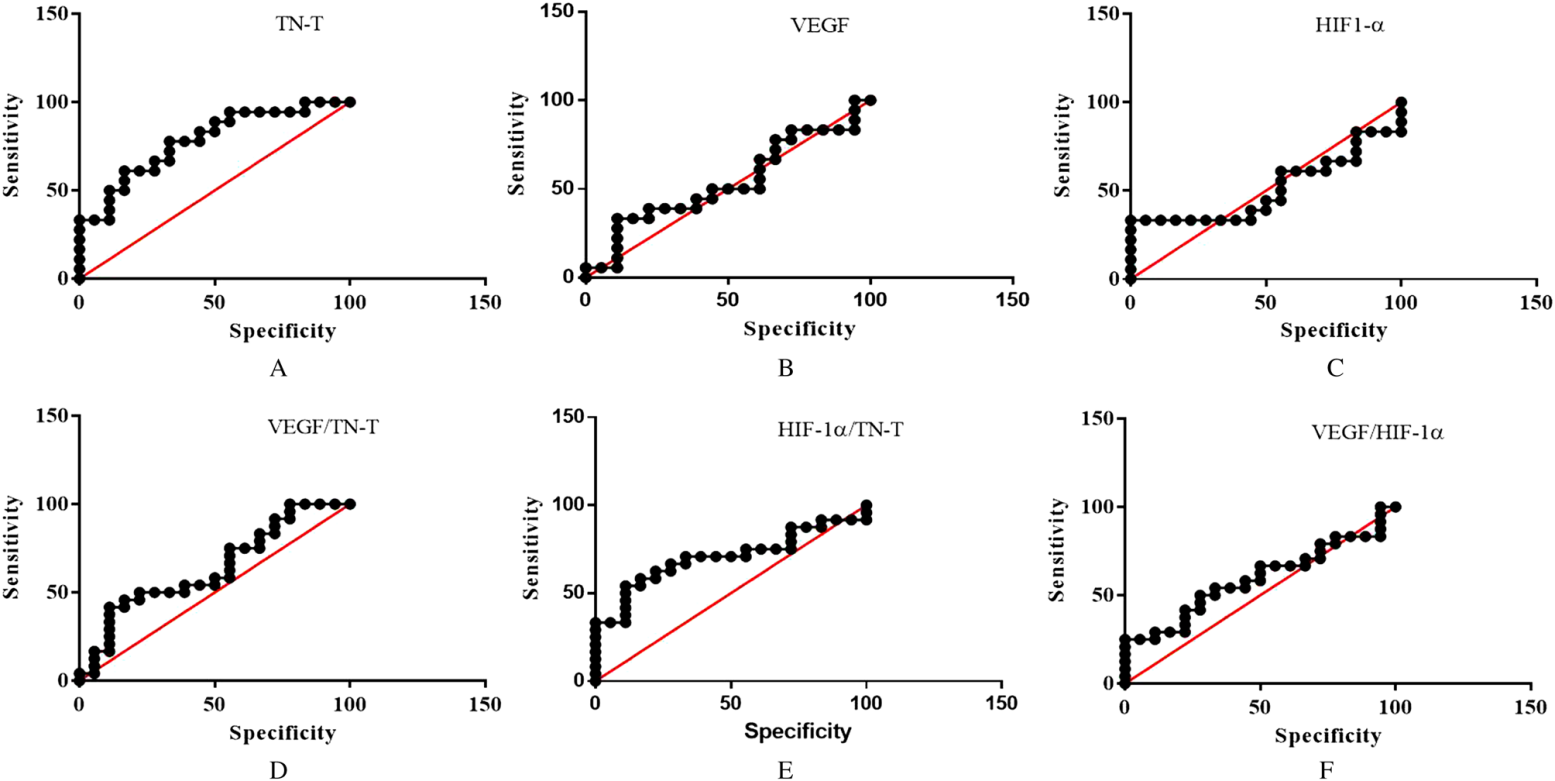
A-F showed the ROC curve of TN-T, VEGF, HIF-1α, VEGF/TN-T, HIF-1α/TN-T and VEGF/HIF-1α respectively.

### Postmortem diagnostic adequacy

We created the ROC curve based on the observation of TN-T, VEGF, HIF-1α, VEGF/TN-T, HIF-1α/TN-T and VEGF/HIF-1α to evaluate the postmortem diagnostic adequacy of them in cantharidin-induced myocardial injury. Figure 4A–F showed the ROC curve of TN-T, VEGF, HIF-1α, VEGF/TN-T, HIF-1α/TN-T and VEGF/HIF-1α respectively, the results suggested that TN-T and HIF-1α/TN-T with higher diagnostic adequacy, and the area under the ROC curve of TN-T and HIF-1α/TN-T were 0.7809(*P* = 0.004), 0.7037 (*P* = 0.0254) respectively.

### Estimation of postmortem interval

**Figure 5:**
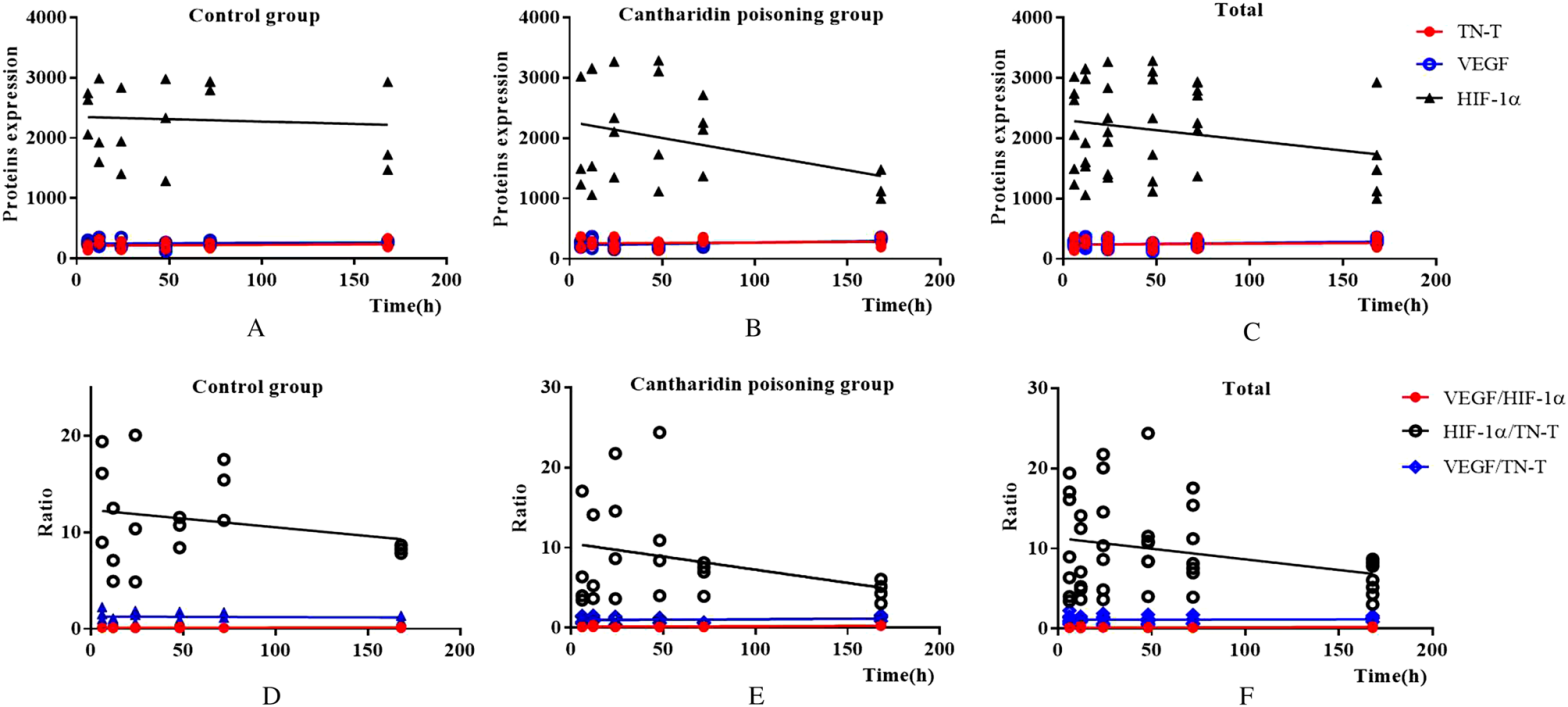
PMI estimation models. (**A**–**C**) showed the linear regression equations between PMI and TN-T, VEGF, HIF-1α in control group, cantharidin poisoning group and total respectively; (**D**–**F**) showed the linear regression equations between PMI and VEGF/TN-T, HIF-1α/TN-T, VEGF/HIF-1α in control group, cantharidin poisoning group and total respectively.

Based on the postmortem changes of the TN-T, VEGF, HIF-1α, VEGF/TN-T, HIF-1α/TN-T and VEGF/HIF-1α in rats cardiac blood, PMI estimation models were constructed based on the linear regression analysis and the results of them showed in Fig. 5. Figure 5A and D showed the linear regression equations were established between PMI and TN-T, VEGF, HIF-1α, VEGF/TN-T, HIF-1α/TN-T, VEGF/HIF-1α in control group and correlations were examined, the equations of them were Y = 0.1384*X + 212.8 (*P* = 0.6143), Y = 0.1214*X + 246.4 (*P* = 0.6581), Y = − 0.7831*X + 2,352 (*P* = 0.7787), Y = − 0.0005427*X + 1.272 (*P* = 0.7959), Y = − 0.01808*X + 12.33 (*P* = 0.3771), Y = 0.0001088* X + 0.1145 (*P* = 0.6306) respectively. The linear regression equations between PMI and TN-T, VEGF, HIF-1α, VEGF/TN-T, HIF-1α/TN-T, VEGF/HIF-1α in experimental were as follows: Y = 0.1389*X + 256.0 (*P* = 0.6275), Y = 0.4487*X + 223.6 (*P* = 0.0785), Y = -5.339*X + 2,270 (*P* = 0.0817), Y = 0.001067*X + 0.9604 (*P* = 0.4600), Y = − 0.03286*X + 10.54 (*P* = 0.1406), Y = 0.0007881*X + 0.1120 (*P* = 0.0135) (Fig. 5B and E). These results suggested that VEGF/HIF-1α with potential value in the estimation of the PMI in experimental group, and when we further studied these data of all rats in the control and expermental (Fig. 5C and F), we also found that evidence for the potential value of VEGF/HIF-1α in the estimation of the PMI, and the regression equation was Y = 0.0004970*X + 0.1131 (*P* = 0.0182).

## Discussion

### Postmortem pathological changes of rat hearts

In forensic identification work, the diagnosis of cantharidin-induced myocardial injury is usually dependent on the histopathologic examination. However, experience shows that pathological changes can change over time due to the autolysis of heart after death. In our present study, myocardial contraction band necrosis, inflammatory cell infiltration and hemorrhage in the subendocardial were observed in cantharidin-treated rats within 48 h after death while these changes were difficult to identify due to the autolysis of myocardium 72 h after death. Consequently, to explore the biomarkers of myocardial injury in rats after cantharidin poisoning may be a solution to the problem.

### Postmortem changes in TN-T, VEGF, HIF-1α, VEGF/TN-T, HIF-1α/TN-T, VEGF/HIF-1α and application for postmortem diagnosis

In our present study, the higher expression of TN-T were found in rats died of cantharidin poisoning and TN-T levels were stable in each time interval after death, which suggested TN-T is a potential postmortem biomarker of mycardial injury induced by cantharidin poisoning. Most previous studies focused on the diagnostic role of postmortem TN-T in patients with acute myocardial infarction or sudden cardiac death and found the elevation of TN-T expression may depend on the severity of ischemic myocardial damage^13,14,25^, which was consisted with the result of the current study. Moreover, our data showed that the TN-T levels in postmortem remained stable for up to 168 h after death, which is over the 48 h reported in previous. However, the accuracy and specificity of TN-T expression in postmortem diagnosis with some controversy^16,18^, and the results of the ROC curve in our study showed that the diagnostic accuracy of TN-T was 78.09% which confirmed previous foundings on TN-T is a specific and useful cardiac biomarker for postmortem diagnosis of myocardial injury.

Variations in total expression of VEGF within 168 h after death between in control and in experimental were not found but it presented an increased trend in our study, the difference of VEGF expression between the two groups was only found in the group of 168 h. The results of previous studes on VEGF expression in human myocardial tissue showed that no statistical differences for expression were detected while VEGF gene expression in body fluids were found at PMI periods of over 12 h; However, our data suggested that the postmortem changes of VEGF protein expression in cardiac blood experienced the process of descending then ascending and the valley value was in the group of 48 h; As mentioned above, we think that the postmortem changes of VEGF may be depends on the cause of death and methods of detection^26,27^.

Our present study indicated that postmortem changes of HIF-1α expression was stable both in control and experimental within 72 h after death. The HIF-1α levels in experimental group was decreased 48 h after death and the differences was found in the group of 72 h compared with control; which was supported by the foundings of Fais P et al. that HIF-1α expression was gradually decreased in samples collected 4–5 days after death and it was not detected 8–9 days after death^28^. So that the postmortem diagnostic role of HIF-1α need more long-term observation over 168 h after death to more accurate conclusions.

The results of postmortem changes in VEGF/TN-T, HIF-1α/TN-T and VEGF/HIF-1α showed that HIF-1α/TN-T may be a cardiac biomarker for postmortem diagnosis of myocardial injury with the 70.37% diagnostic accuracy; And we also found that all of the ratios were stable in control after death in each time interval; while the ratios of VEGF/TN-T and VEGF/HIF-1α were increased over 72 h after death in experimental, and the postmortem diagnostic role of them also need more long-term observation to evaluate. What is important, we found that the study on diagnostic role of the ratios is not mentioned in previous.

In this section, we found that TN-T and HIF-1α/TN-T are cardiac biomarkers for postmortem diagnosis of cantharidin-induced myocardial injury, and other biomarkers have potiencial value in the postmortem diagnosis in different time intervals after death. These interesting results may provide forensic pathologists with a reference to the postmoterm diagnosis of myocardial injury; And we hope that with more long-term observation and the verification of these biomarkers in other types of myocardial injury, the biomarkers in our study could be used in the postmorterm diagnosis of myocardial injury in future.

### Association between TN-T, VEGF, HIF-1α, VEGF/TN-T, HIF-1α/TN-T, VEGF/HIF-1α and PMI

PMI estimation remains a challenge in the forensic identification due to the lack of efficient methods. As we know, forensic protein technology has been used to estimate the PMI for many years; and many previous studies focused on the postmortem degradation of TN-T and its association with PMI. Kumar et al.^29^ found that the degradation of TN-T in cardiac tissue samples with respect to time in deaths of mycardical infraction, burn, electrocution, control, poisoning and asphyxia. However, in our present study, the postmortem TN-T expression in cardiac blood both in control group and experimental group were stable and regression analysis results revealed no significant relationship between TN-T and PMI. There are multiple possible reasons for discrepant results between previous study and current study, such as the rate of fragmentation of TN-T in different samples, the cause of death, temperature, and so on^13–18,29^.

Our data in present study indicated that the postmortem changes of VEGF both in control and experimental experienced the process of descending then ascending and especially in experimental groups that the the levels of VEGF started to increase after 12 h PMI and decreased with PMI up to 48 h, then increased linearly with PMI up to 168 h; and results of previous studies^27,30^ also showed that the time-course of VEGF expression with tissue-specific differences. So that the regression analysis results revealed no significant relationship between VEGF and PMI. Based on the present data, the model uncover the non-linear relationship between VEGF and PMI can be built in further study which will provide a reference for the PMI estimation in forensic identification.

Regression analysis results revealed no significant relationship between HIF -1α and PMI within the 168 h after death both in control and experimental. In our present study, we found that the postmortem changes of HIF-1α was consistent with previous study that HIF-1α expression was gradually decreased 4–5 days after death^28^; However, HIF-1α protein expression as a new marker for PMI estimation in human gingival tissue was confirmed in previous study and the results of the study suggested HIF1α is a promising biomarker for distinguishing deaths that have occurred within 1 week from those that occurred more than 1 week ago; So that much more long-term observation of HIF-1α expression in our further study will help us to explain the difference between previous study and our current study.

Regression analysis results revealed a significant relationship between VEGF/HIF-1α and PMI while no significant relationships between VEGF/TN-T and PMI, HIF-1α/TN-T and PMI in experimental group. These data indicated that the ratio of VEGF/HIF-1α appears to be a useful method of estimating the PMI up to 168 h in forensic identification. But regression analysis results revealed no significant relationships between VEGF/TN-T and PMI, HIF-1α/TN-T and PMI, VEGF/HIF-1α and PMI.

Based on the above founding, we further analyzed the association between TN-T, VEGF, HIF-1α, VEGF/TN-T, HIF-1α/TN-T, VEGF/HIF-1α and PMI by using linear regression analysis and the results revealed a significant relationship between VEGF/HIF-1α and PMI regardless of cause of death. This finding is an encouraging discovery really immediately that VEGF/HIF-1α promises to be a biomarker to estimate PMI regardless of cause of death; However, this finding needs to be verified by larger sample in further study.

## Conclusions

In summary, these results in present study suggested that plasma levels of TN-T and HIF-1α/TN-T are cardiac biomarkers with higher diagnostic accuracy for postmortem diagnosis of cantharidin-induced myocardial injury; VEGF/HIF-1α promises to be a biomarker for PMI estimation. Although further verification studies are required, these results seem to be encouraging and demonstrate that these biomarkers may have a high potential for being a useful tool in postmortem diagnosis of cantharidin-induced myocardial injury, and PMI estimation.

## References

[1] Worthley LI (2002). Clinical toxicology: part II. Diagnosis and management of uncommon poisonings. Crit. Care Resusc..

[2] Hundt HK, Steyn JM, Wagner L (1990). Post-mortem serum concentration of cantharidin in a fatal case of cantharides poisoning. Hum. Exp. Toxicol..

[3] Zhang Y, Zhou X, Zhang J, Guan C, Liu L (2018). Cantharides poisoning: A retrospective analysis from 1996 to 2016 in China. Regul. Toxicol. Pharmacol..

[4] Marcovigi P, Leoni S, Calbi G, Valtancoli E, Ravaglia G (1995). Acute poisoning caused by cantharidin ingestion for aphrodisiac purposes. A clinical case. Minerva Anestesiol..

[5] Polettini A, Crippa O, Ravagli A, Saragoni A (1992). A fatal case of poisoning with cantharidin. Forensic Sci. Int..

[6] Cotovio P, Silva C, Guedes Marques M, Ferrer F, Costa F, Carreira A, Campos M (2013). Acute kidney injury by cantharidin poisoning following a silly bet on an ugly beetle. Clin. Kidney J..

[7] Tagwireyi D, Ball DE, Loga PJ, Moyo S (2000). Cantharidin poisoning due to "Blister beetle" ingestion. Toxicon.

[8] Wijerathne BTB (2017). Blister mystery. Wilderness Environ. Med..

[9] Chen JH, Inamori-Kawamoto O, Michiue T, Ikeda S, Ishikawa T, Maeda H (2015). Cardiac biomarkers in blood, and pericardial and cerebrospinal fluids of forensic autopsy cases: a reassessment with special regard to postmortem interval. Leg. Med..

[10] Wang H, Ma J, Xu H, Lyu Y, Tao L, Li W, Zeng Y, Ma K, Xiao B, Chen L (2019). Early postmortem interval (EPMI) estimation using differentially expressed gene transcripts. Leg. Med..

[11] Choi KM, Zissler A, Kim E, Ehrenfellner B, Cho E, Lee SI, Steinbacher P, Yun KN, Shin JH, Kim JY (2019). Postmortem proteomics to discover biomarkers for forensic PMI estimation. Int. J. Legal Med..

[12] Hartmann F, Kampmann M, Frey N, Muller-Bardorff M, Katus HA (1998). Biochemical markers in the diagnosis of coronary artery disease. Eur. Heart J..

[13] Barberi C, van den Hondel KE (2018). The use of cardiac troponin T (cTnT) in the postmortem diagnosis of acute myocardial infarction and sudden cardiac death: a systematic review. Forensic Sci. Int..

[14] Gonzalez-Herrera L, Valenzuela A, Ramos V, Blazquez A, Villanueva E (2016). Cardiac troponin T determination by a highly sensitive assay in postmortem serum and pericardial fluid. Forensic Sci. Med. Pathol..

[15] Zhu BL, Ishikawa T, Michiue T, Li DR, Zhao D, Kamikodai Y, Tsuda K, Okazaki S, Maeda H (2006). Postmortem cardiac troponin T levels in the blood and pericardial fluid. Part 2: analysis for application in the diagnosis of sudden cardiac death with regard to pathology. Leg. Med. (Tokyo).

[16] Remmer S, Kuudeberg A, Tonisson M, Lepik D, Vali M (2013). Cardiac troponin T in forensic autopsy cases. Forensic Sci. Int..

[17] Nowak A, Nowak S, Chowaniec C, Wojnicz R (2012). Troponin in forensic medicine. Arch. Med. Sadowej Kryminol..

[18] Rahimi R, Dahili ND, Anuar Zainun K, Mohd Kasim NA, Md NS (2018). Post mortem troponin T analysis in sudden death: Is it useful?. Malays. J. Pathol..

[19] Liu L, Zhang YG, Deng WN, Liu Y, Deng J, Zhou YW, Haung GZ (1993). Study on pathological change of acute cantharidies poisoning. Chin. J. Forensic Med..

[20] Forsythe JA, Jiang BH, Iyer NV, Agani F, Leung SW, Koos RD, Semenza GL (1996). Activation of vascular endothelial growth factor gene transcription by hypoxia-inducible factor 1. Mol. Cell Biol..

[21] Yamakawa M, Liu LX, Date T, Belanger AJ, Vincent KA, Akita GY, Kuriyama T, Cheng SH, Gregory RJ, Jiang C (2003). Hypoxia-inducible factor-1 mediates activation of cultured vascular endothelial cells by inducing multiple angiogenic factors. Circ. Res..

[22] Greer SN, Metcalf JL, Wang Y, Ohh M (2012). The updated biology of hypoxia-inducible factor. EMBO J..

[23] Michaud SE, Ménard C, Guy LG, Gennaro G, Rivard A (2003). Inhibition of hypoxia-induced angiogenesis by cigarette smoke exposure: impairment of the HIF-1alpha/VEGF pathway. FASEB J..

[24] Youyou Z, Yalei Y, Jie Z, Chuhuai G, Liang L, Liang R (2020). Molecular biomarkers of cantharidin-induced cardiotoxicity in Sprague-Dawley rats: Troponin T, vascular endothelial growth factor and hypoxia inducible factor-1α. J. Appl. Toxicol..

[25] Cao Z, Zhao M, Xu C, Zhang T, Jia Y, Wang T, Zhu B (2019). Diagnostic roles of postmortem cTn I and cTn T in cardiac death with special regard to myocardial infarction: a systematic literature review and meta-analysis. Int. J. Mol. Sci..

[26] Gonzalez-Herrera L, Valenzuela A, Marchal JA, Lorente JA, Villanueva E (2013). Studies on RNA integrity and gene expression in human myocardial tissue, pericardial fluid and blood, and its postmortem stability. Forensic Sci. Int..

[27] Thaik-Oo M, Tanaka E, Tsuchiya T, Kominato Y, Honda K, Yamazaki K, Misawa S (2002). Estimation of postmortem interval from hypoxic inducible levels of vascular endothelial growth factor. J. Forensic Sci..

[28] Fais P, Mazzotti MC, Teti G, Boscolo-Berto R, Pelotti S, Falconi M (2018). HIF1α protein and mRNA expression as a new marker for post mortem interval estimation in human gingival tissue. J. Anat..

[29] Kumar S, Ali W, Bhattacharya S, Verma AK (2016). The effect of elapsed time on cardiac troponin-T (cTnT) degradation and its dependency on the cause of death. J. Forensic Leg. Med..

[30] Zhao D, Ishikawa T, Quan L, Li DR, Michiue T, Yoshida C, Komatu A, Chen JH, Zhu BL, Maeda H (2008). Tissue-specific differences in mRNA quantification of glucose transporter 1 and vascular endothelial growth factor with special regard to death investigations of fatal injuries. Forensic Sci. Int..

