# Author Correction: Biomarkers of myocardial injury in rats after cantharidin poisoning: Application for postmortem diagnosis and estimation of postmortem interval

**DOI:** 10.1038/s41598-020-74782-7

**Published:** 2020-10-20

**Authors:** Youyou Zhang, Yalei Yu, Jie Zhang, Chuhuai Guan, Liang Liu, Liang Ren

**Affiliations:** Department of Forensic Medicine, Tongji Medical College, Huazhong University of Science and Technology, Wuhan, 430030 China

Correction to: *Scientific Reports* 10.1038/s41598-020-69118-4, published online 21 July 2020

In the original version of this Article, Youyou Zhang was incorrectly listed as a corresponding author. The correct corresponding authors for this Article are Liang Liu and Liang Ren.

Additionally, the original Article contained two email addresses for Liang Liu. The correct email address is below.

Correspondence and requests for materials should be addressed to 869974299@qq.com for Liang Liu, or 36918280@qq.com for Liang Ren.

This error has now been corrected in the HTML and PDF versions of this Article.

